# Is There an Association between the Use of Epidural Analgesia during Labor and the Development of Autism Spectrum Disorder in the Offspring?—A Review of the Literature

**DOI:** 10.3390/ijerph19127202

**Published:** 2022-06-12

**Authors:** Joanna Weronika Król, Paweł Jan Stanirowski, Natalia Mazanowska, Agata Majewska, Mirosław Wielgoś, Dorota Bomba-Opoń

**Affiliations:** 1st Department of Obstetrics and Gynecology, Medical University of Warsaw, Starynkiewicza Sq. 1/3, 02-015 Warsaw, Poland; stanirowski@gmail.com (P.J.S.); natalia.mazanowska@gmail.com (N.M.); majewska.agata@gmail.com (A.M.); miroslaw.wielgos@wum.edu.pl (M.W.); dorota.bomba-opon@wum.edu.pl (D.B.-O.)

**Keywords:** autism spectrum disorders, epidural analgesia, pregnancy

## Abstract

Autism spectrum disorders (ASDs) are multifactorial and complex neurodevelopmental conditions usually diagnosed in the early childhood. The etiology of ASDs is commonly described as a genetic predisposition combined with an environmental impact. As a result of broadening of the diagnostic criteria the prevalence of ASDs has been increasing worldwide and the search for the modifiable factors is still on-going. Epidural analgesia (ELA) provides effective pain relief during labor and is currently the most preferred method of anesthesia during the delivery. The safety of the procedure is well-discussed and documented; nonetheless, in 2020 a single population-based study indicated an association between the use of ELA during labor and newborn risk of ASD development, which led to widespread concern. To explore the possible association between the ELA and ASD occurrence in the offspring several studies in different countries have been conducted to date. In this review we aimed to summarize the current state of knowledge concerning the association between the use of epidural analgesia during labor and risk of ASD. In conclusion, the literature review indicates that there is no significant association.

## 1. Introduction

Autism spectrum disorders (ASDs) are multifactorial and complex neurodevelopmental conditions usually diagnosed in early childhood. The main diagnostic criteria include the presence of persistent deficits in social communication and social interaction across multiple contexts [1]. The etiology of ASDs is commonly described as a genetic predisposition combined with an environmental impact [2]. The overall recurrence between siblings is approximately 25%, whereas twins demonstrate up to 30% to 80% concordance rate depending on the dizygotic or monozygotic origin, respectively [3,4]. The environmental risk factors include advanced parental age, prematurity, short-interval pregnancies, maternal obesity, maternal diabetes, and birth complications involving hypoxia or trauma [5]. The prevalence of ASDs has been increasing worldwide e.g., the average prevalence before 1980 in Asia was approximately 1.9 cases per 10,000 rising to 14.8 between 1980 and 2010 [6,7]. Although this rise is partly believed to be connected to the broadening of diagnostic criteria, the search for the modifiable factors is still on-going. 

Epidural analgesia (ELA) provides effective pain relief during labor and is currently the most preferred method of anesthesia during delivery [8]. The safety of the procedure is well-discussed and documented with the Cochrane review from 2018 stating that ELA with low concentration of local anesthetic has no adverse impact on the proportions of Caesarean section, long-term backache, or neonatal outcomes [9,10]. Nonetheless, in 2020 Qiu et al. published the population-based study indicating an association between the use of ELA during labor and newborn risk of ASD, which led to widespread concern [11]. Since the ELA is commonly acknowledged as a standard method of labor analgesia, following the above-mentioned publication, several of the major obstetrics’ and anesthesiologists’ societies issued statements emphasizing both the lack of plausible mechanisms to support a causal link between the use of epidural and risk of ASD, and the difference between association and causation [12,13]. To explore the possible connection between ELA and ASD occurrence in the offspring, several studies in different countries have been conducted to date. 

In this review we aimed to summarize the current state of knowledge concerning the association between the use of epidural analgesia during labor and risk of ASD, and therefore to evaluate if the individual risk of autism spectrum disorders should be considered during the decision whether to use neuraxial labor anesthesia. 

## 2. Materials and Methods

**Figure 1:**
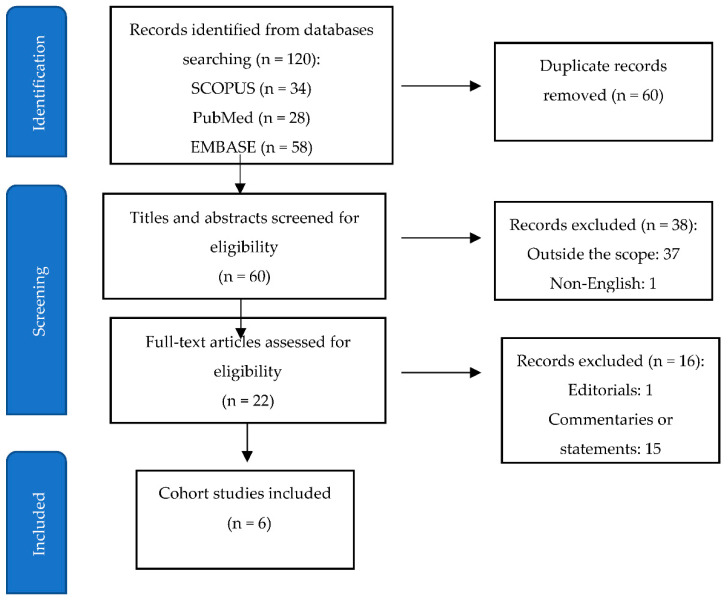
Flow-chart displaying the selection process.

A literature search was conducted to identify relevant studies addressing the question of the possible association between the ELA and the risk of ASD development in the offspring. Scopus, PubMed and EMBASE databases were searched for all articles published until March 2022, using the key words: epidural anesthesia, neuraxial labor anesthesia, labor, autism. The search yielded 120 results (Figure 1) of which 98 articles were excluded: 60 duplicates, 37 articles were outside the scope of the investigation, and one article was only available in German. Of the remaining 22 articles in English—one was editorial and six were original papers, with the rest being the statements or commentaries to the study by Qiu et al. These six cohort studies constitute the basis of this review. 

## 3. Results

The reviewed articles were published between 2020 and 2022 and originated in three different countries—United States, Canada and Denmark—and all were population-based retrospective cohort studies. Table 1 summarizes studies and follow-up periods, as well as classifications of outcomes of the reviewed publications. 

The study by Qiu et al. was the first to investigate the relationship between the use of ELA and the development of autism in the offspring. It included 147,895 singleton children born by vaginal delivery at 28 to 44 weeks’ gestation in Southern California, of which 109,719 (74.2%) were exposed to ELA. A total of 2524 children received a diagnosis of ASD during the follow-up period: 2039 (1.9%) in the ELA group and 483 (1.3%) in the non-ELA group. The authors concluded that maternal exposure to ELA was associated with a 37% increase in the risk of ASD after adjusting for potential cofounders. In addition, longer duration of epidural exposure occurred to be an independent risk factor of ASD development. The exposure to ELA of less than 4 h, 4 to 8 h and of more than 8 h was associated with the 33%, 35% and 46% greater risk of ASD compared with the unexposed group, respectively. 

In another study Wall-Weiler et al. analyzed the data from health care databases in Manitoba, Canada from 2005 to 2016 [14]. Their study included 123,175 singleton offspring born by vaginal birth of which 47,011 (38.2%) were exposed to ELA. The cumulative risk of ASD development was 2.1% and 1.7% for offspring exposed or unexposed to ELA, respectively. Similar to the study by Qiu et al. a significant association between ELA and an offspring risk of ASD was shown in the unadjusted analysis (Hazard Ratio (HR) 1.25; 95% CI, 1.15–1.36), however, this interrelation was not observed after adjusting for a large set of pre-pregnancy, sociodemographic and perinatal confounders including maternal age, parity, pre-existing and gestational diabetes mellitus, hypertension, induction of labor, premature rupture of membranes, offspring sex etc. (aHR 1.08; 95% CI, 0.97–1.20). Importantly, results from the additional sibling cohort analysis that account for unmeasured stable familial variables (e.g., parental ASD diagnosis and potential genetic factors) matched those obtained in principal models and demonstrated no significant association (HR 0.97; 95% CI, 0.78–1.22). 

The other two studies from Denmark used the same data sources and classifications for ELA and ASD but reported different adjusted hazard ratios [16,17]. The first study conducted by Mikkelsen et al. identified all live-born children in Denmark between January 2006 and December 2013 with the offspring follow-up up to December 2017. Among 92,900 children exposed to ELA 1409 (1.5%) were diagnosed with ASD compared with 5019 (1.3%) of 386,278 unexposed, which accounted for an unadjusted HR of 1.29 (95% CI, 1.21–1.37). In this primary analysis ELA was not associated with a significantly higher risk than no exposure (aHR 1.05; 95% CI, 0.98–1.11). Additionally, researchers conducted secondary analysis utilizing a within-mother design and including only children of mothers with both exposure and non-exposure to ELA in different deliveries (n = 59 154, 12.3%). Likewise, the analysis did not confirm that exposure to ELA was significantly associated with the offspring ASD (aHR 1.05; 95% CI, 0.90–1.21). The second study conducted by Ren et al. included 624,952 live-born singletons delivered vaginally in Denmark between 2005 and 2016 and focused not only on the association between ELA and ASD but also on the risk of other neurodevelopmental disorders in exposed offspring e.g., attention-deficit hyperactivity disorder (ADHD), intellectual disability (ID) and epilepsy. Cumulative incidence of ASD in exposed offspring reached 3.2% over 14 years and in unexposed 2.6%. After adjustment for potential confounders an 11% increase in the risk of ASD was noted in the offspring exposed to ELA (aHR 1.11; 95% CI, 1.04–1.18). Similar to the study by Qiu et al. the authors analyzed the impact of ELA duration on ASD occurrence, but the results did not reveal any significant association. It is worth noting that although the full-cohort analysis demonstrated a slightly higher risk of ASD with ELA, the sibling-matched analysis showed no such association. This can suggest that the cohort analyses might have been confounded by time-stable environmental, unknown genetic factors and social confounders that were better accounted for in the sibling-matched analysis. In addition, the authors observed that ELA was associated with specific developmental disorder (SDD) (HR 1.12; 95% CI, 1.03–1.22), but not with ADHD (HR 0.98; 95% CI, 0.92–1.03), ID (HR 0.98; 95% CI, 0.85–1.14), or epilepsy (HR 0.89; 95% CI, 0.79–1.00). Interestingly, once again the association between ELA and SDD disappeared in the sibling-matched analysis. The observed difference in adjusted hazard ratios between the two Danish studies may be explained by different selection of confounders and statistical approaches. Otherwise, both studies came to the same conclusions and showed that the causal effect of ELA on ASD development in the offspring cannot be supported.

The last two studies by Hanley et al. and the most recently published by Straub et al. demonstrated a small increase in the risk of ASD in the offspring exposed to ELA [15,18]. The first study included 388,254 term singleton vaginal deliveries in British Columbia, Canada between 1 April 2000, and 31 December 2014, with the follow-up up to 31 December 2016. 111,480 (28.7%) children were exposed to epidural analgesia from whom 1710 (1.53%) were diagnosed with ASD compared to 3482 (1.26%) among the 276,774 unexposed. Authors reported HR of 1.32 (95% CI, 1.24–1.40) in the unadjusted analysis. Although after adjusting for maternal sociodemographic characteristics ELA remained significantly associated with an increased risk of ASD, it became attenuated following further adjustments for maternal condition during pregnancy (HR 1.12; 95%, CI 1.05–1.20), as well as labor and delivery conditions (HR 1.09; 95% CI, 1.00–1.15). Like previous studies the within-mother matched analysis and the sibling cohort showed no statistically significant association between epidural analgesia and ASD. In the second study by Straub et al. authors used both public and private health care databases from United States. In total they examined 1.6 million registered vaginal births from 2005 to 2015 with 998,099 (62.1%) exposed to ELA. In the publicly insured cohort cumulative incidence of ASD by the age of 10 among children with ELA exposure reached 1.93% (95% CI, 1.73–2.13%) versus 1.64% (95% CI, 1.51–1.76%) among those without, whereas in the privately insured cohort the respective incidence was lower and reached 1.33% (95% CI, 1.19–1.46%) versus 1.19% (95% CI, 0.99–1.38%). This corresponded to an unadjusted pooled HR of 1.06 (95% CI, 1.00–1.12). When adjusted, the HRs for the publicly and privately insured cohorts were 1.07 (96% CI, 0.99–1.17) and 1.06 (95% CI, 0.94–1.18), respectively, which corresponded to a pooled HR of 1.07 (95% CI, 1.00–1.14). Simultaneously, the authors conducted a meta-analysis using the data from four original studies, which gave a pooled adjusted HR of 1.10 (95% CI, 1.06–1.13) with a *P* value of 0.001 suggesting significant between-study heterogeneity. Although both above-mentioned studies observed an increase in the risk of ASD in the offspring exposed to ELA, given the probability of residual confounding, they do not provide strong supporting evidence for the association. 

## 4. Discussion

In this review of literature, we aimed to assess if there is an association between the use of epidural analgesia during labor and risk of ASD development. To the best of our knowledge, it is the most actual review of the literature addressing the topic published to date.

The research by Qiu et al. which reported 37% increase in the risk of ASD development in the offspring exposed to ELA is so far the only study to prove significant association. The later published five cohort studies reported HRs between 1.05 and 1.11 with pooled adjusted HR of 1.07 reflecting a lack of significant interrelation [18]. The study by Qiu et al. was widely criticized and many limitations were identified by the experts. For example, Hanley et al. raised concern about using diagnostic codes for the identification of children with ASD as the most recent research shows that they have low validity when identifying the disorder, and thus the study was susceptible to bias [19,20]. The authors also focused on the lack of the maternal and family history of ASD in the confounding factors and the social and medical differences between the studied populations i.e., women who chose to use ELA had more biological risk factors of ASD including advanced age, preeclampsia, diabetes and obesity. These limitations were also emphasized in other commentaries to the study [21,22,23].

Another important remark was made by Khashan who pointed to the lack of accounting for the indications for the use of ELA as the potential confounder [24]. As an example, he used the fact that maternal anxiety and depression are both associated with the use of ELA and the increased risk of ASD. Therefore, the reported association between ELA and ASD could be the result of bias. Similar concern was also raised by Kern-Goldberg et al. and Lee et al. along with the lack of discussion about the labor complications [22,25].

The latest editorial published by Carrier et al. drew attention to the statistical techniques used by Qiu et al. [21]. The Inverse Probability of Treatment Weighting analysis assumes that all confounders of the association of interest are known, adequately measured and included in the model. As mentioned before, the study failed to address some of the important confounders e.g., maternal and family history of ASD, and thus it could provide biased estimations that cannot suggest causation. A similar issue was also discussed by Saito et al. in the letter to the editor [26].

The final concern was expressed by Lee et al. who pointed out that the observations from a single hospital center may not be generalizable, in particular given the critical contribution of genetic and environmental factors to ASD [22]. 

Some of the above-mentioned concerns were addressed by Qiu et al. who in the reply to the letters to editors written by Lee et al. and Kern-Goldberg et al. stated that the lack of the known mechanism in which anesthetics could trigger the development of ASD in neonates does not exclude its existence [27]. Furthermore, it is worth mentioning that other researchers also used diagnostic codes to identify offspring ASD, which made their studies as susceptible to error as the one by Qiu et al. Nevertheless, the most discussed reason for the difference in the outcome is the failure to address some of important confounders e.g., maternal and family history of ASD or labor complications, all making the study vulnerable to bias. 

## 5. Conclusions

To conclude, the current literature review indicates that there is no significant association between the use of epidural anesthesia during labor and later development of autism spectrum disorder in the offspring. Therefore, the individual risk of ASD should not be considered while deciding on the pain management method during delivery. 

## Figures and Tables

**Table 1:** Summary of studies included in the review.

	Qiu et al. [11]	Wall-Weiler et al. [14]	Hanley et al. [15]	Mikkelsen et al. [16]	Ren et al. [17]	Straub et al. [18]
Study Period	1 January 2008–31 December 2015 Follow-up: from 1 year of age till clinical diagnosis of ASD, end of healthcare plan, death of the child or study end date 31 December 2018	1 April 2005–31 March 2016 Follow-up: from 18 months of age until 1 April 2019 or censored by death or emigration	1 April 2000–31 December 2014 Follow-up: until clinical diagnosis of ASD, death or study end date 31 December 2016	1 January 2006–31 December 2013 Follow-up: from 1 year of age till 31 December 2017 or censored by death, emigration, diagnosis of disease inherently linked to autism	1 January 2005-31 December 2016 Follow-up: for ASD from 1 year of age till death, emigration, diagnosis of ASD or 31 December 2018	2005–2014 data from Medicaid Analytic eXtract (MAX) 2005–2015 data from IBM Health MarketScan Research Database (MarketScan) Follow-up: until ASD diagnosis, end of insurance or end of study period
Study Population	147,895 singleton vaginal deliveries in Southern California, USA	123,175 singleton vaginal deliveries in Manitoba, Canada	388,254 term singleton vaginal deliveries in British Columbia, Canada	479,178 all liveborn offspring in Denmark	624,952 live singleton vaginal or intrapartum Caesarean delivieries in Denmark	1,607,579 vaginal deliveries registered in MAX or MarketScan in USA
Exposed to ELA (%)	109,719 (74.2%)	47,011 (38.2%)	111,480 (28.7%)	92,900 (19.4%)	116,296 (18.6%)	998,099 (62.1%)
Classification/Source for ELA	Electronic patient records	Hospital Abstracts Data set	British Columbia Perinatal Data Registry	Danish Patient Registry	Danish Patient Registry	MAX or MarketScan Patient Data
Classification/Source for ASD	ICD-9 codes or KPSC equivalent codes for autistic disorders, Asperger syndrome or pervasive development disorder	ICD-9-CM or ICD-10-CA codes for autism disorders, Asperger syndrome or pervasive development disorder	Diagnoses data made by trained practitioner within the British Columbia Autism Assessment Network or private practitioners in Britsh Columbia.	ICD-10 codes for autistic disorder, atypical autism, Asperger syndrome or pervasive development disorders	ICD-10 codes for childhood autism, atypical autism, pervasive developmental disorders and unspecified pervasive disorders	ICD-9 codes for pervasive developmental disorder (excluding childhood disintegrative disorder); diagnosed at least twice at 1 year or older
Total Number of Offspring with ASD (%)	2524 (1.9%)	2257 (1.8%)	5192 (1.3%)	6428 (1.3%)	7671 (1.2%)	5177 (0.3%)
Offspring with ASD in non-ELA group (%)	485 (1.3%)	1272 (1.7%)	3482 (1.26%)	5019 (1.3%)	6023 (1.2%)	2155 (0.4%)
Offspring with ASD in ELA group (%)	2039 (1.9%)	985 (2.1%)	1710 (1.53%)	1409 (1.5%)	1648 (1.4%)	3022 (0.3%)
Unadjusted Hazard Ratio (HR)	1.48 (95% CI, 1.34–1.65)	1.25 (95% CI, 1.15–1.36)	1.32 (95% CI, 1.24–1.40)	1.29 (95% CI, 1.21–1.37)	1.38 (95% CI, 1.31–1.46)	1.06 (95% CI, 1.00–1.12)
Fully Adjusted HR	1.37 (95% CI, 1.22–1.53)	1.08 (95% CI, 0.97–1.20)	1.09 (95% CI, 1.00–1.15)	1.05 (95% CI, 0.98–1.11)	1.11 (95% CI, 1.04–1.18)	1.07 (95% CI, 1.00–1.14)
HR in Siblings Analysis if done	Not done	0.97 (95% CI, 0.78–1.22)	1.10 (95% CI, 0.99–1.20)	Not done	0.95 (95% CI, 0.76–1.18)	Not done

## Data Availability

Not applicable.
